# Understanding Activation Patterns in Shared Circuits: Toward a Value Driven Model

**DOI:** 10.3389/fnhum.2018.00180

**Published:** 2018-05-07

**Authors:** Lisa Aziz-Zadeh, Emily Kilroy, Giorgio Corcelli

**Affiliations:** ^1^Brain and Creativity Institute, University of Southern California, Los Angeles, CA, United States; ^2^Division of Occupational Science and Occupational Therapy, University of Southern California, Los Angeles, CA, United States; ^3^Department of Economics, University of Southern California, Los Angeles, CA, United States

**Keywords:** mirror neuron system, motor learning, social cognition, shared neural networks, value-based decision making

## Abstract

Over the past decade many studies indicate that we utilize our own motor system to understand the actions of other people. This mirror neuron system (MNS) has been proposed to be involved in social cognition and motor learning. However, conflicting findings regarding the underlying mechanisms that drive these shared circuits make it difficult to decipher a common model of their function. Here we propose adapting a “value-driven” model to explain discrepancies in the human mirror system literature and to incorporate this model with existing models. We will use this model to explain discrepant activation patterns in multiple shared circuits in the human data, such that a unified model may explain reported activation patterns from previous studies as a function of value.

## Introduction

Understanding other people’s actions and feelings is an essential component of successful social interactions. Recent neuroscience data indicate that the neural mechanisms involved in processing one’s own actions, sensations and emotions are also involved in perceiving and understanding the actions, sensations and emotions of others (Gallese and Goldman, 1998; Keysers et al., 2004, 2010; Keysers and Gazzola, 2009). These “shared circuits” are thought to constitute pre-reflective processes in social cognition, involving automatic and intuitive levels of representation (Coricelli, 2005). Such shared circuits include the mirror neuron system (MNS), the pain matrix and the somatosensory cortices.

The MNS (inferior frontal gyrus [IFG], ventral premotor cortex [vPMC] and posterior parietal cortex [PPC]) responds both when one executes an action and when one observes someone else make the same action (e.g., opening a bottle of champagne and watching someone else perform the action; Rizzolatti and Craighero, 2004). Data also indicate that some mirror neurons may also respond to the sounds of actions (Kohler, 2002). Because observing others activates one’s own motor systems, it is thought that the MNS is important for action and social understanding. That is, part of how we may understand other people’s actions and intentions is by simulating their actions onto our own motor representations (Keysers et al., 2004).

Since the discovery of the MNS, it has been suggested that other brain regions might also be active both for processing one’s own experiences as well for processing the experiences of other people. For example, there is evidence for a shared neural system for processing disgust, where human subjects use the same neural regions in the anterior insula for the physical experience of disgust as well as for perceiving another person experience disgust (facial expressions), has also been shown (Wicker et al., 2003). A similar social mirroring mechanism is thought to exist for emotion and pain processing as well as somatosensation. Previous research indicates that we process other people’s pain by activating the neural systems that processes pain in our own bodies. This “pain matrix” includes the insula, anterior and middle cingulate gyrus and somatosensory cortices (Singer et al., 2004; Avenanti et al., 2005; Jackson et al., 2006; Bufalari et al., 2007; Di Cesare et al., 2015), though it has been suggested that this network responds to a variety of salient stimuli (Lannetti and Mouraux, 2010). In addition, watching another person being touched (e.g., watching a snake slither up another person’s leg or being brushed up against by a cat) activates our own secondary somatosensory cortices (Keysers et al., 2004; Meyer, 2011). Keysers et al. (2004) reported that observing another person’s leg being stroked activated the secondary somatosensory cortex (SII) bilaterally, the same as when the person was touched themselves. Such “shared neural circuits” involved in processing one’s own experiences as well as the experiences of other people, might be an important neural basis of social cognition.

### Discrepancy in Research Findings Regarding Activation of Shared Neural Circuits

The idea that we utilize our own sensorimotor representations to process and understand other people has been the focus of much recent research. However, there has been a great deal of discrepant findings in what drives activity in these shared neural circuits (e.g., MNS, somatosensory cortices, pain matrix, emotion-related brain regions). Some research groups have shown increased activity in shared circuits for observing liked individuals (Singer et al., 2006) or individuals more similar to the self (Xu et al., 2009) while other studies show the opposite (Fox et al., 2013; Losin et al., 2014). Some groups find more activity for observing actions for which one has expertise (Raichle et al., 2001; Cross et al., 2006, 2009; Beudel et al., 2011; Liew et al., 2011; Gardner et al., 2015), while other groups find that novelty drives activation patterns (Cross et al., 2012; Aziz-Zadeh, 2013; Grossmann et al., 2013; Liew et al., 2013; Tipper et al., 2015). These discrepant findings (discussed further in “Relation of the Value Model to Human Data” section) not only raise important questions about what drives activity in shared neural circuits, but also their function.

## Previous Models

One model that has previously been put forward to better understand the function of the MNS and its activation patterns utilizes the notion of predictive modeling. In this model, Kilner et al. (2007) utilize largely established forward or generative models that are critical to motor control (Miall and Wolpert, 1996; Wolpert et al., 2003; Kilner et al., 2007; Neal and Kilner, 2012). They extend the same model for action observation. The predictive coding account posits that the MNS is involved in creating predications of other people’s actions (Gallese and Goldman, 1998), the context (Liepelt et al., 2009), or their physical body (Buccino et al., 2004). Following an empirical Bayes inference, the model states that our prior expectations of an action have an associated standard deviation. A predication error is generated by the comparison between the predicted action and the actual observed action. Thus, they theorize that predictive coding may provide a computational framework for inferring the causes of sensory information. In the case of action observation, causes may include goals, intentions and motor commands and sensory inputs may include observed kinematics. Thus, when we observe someone swing a tennis racket and hit a ball, we may use the same models that we use to perform the action ourselves to infer motor commands and kinematics from the observed actions of other people. A discrepancy in prediction error would result in greater MNS activation, which may reflect increased demands to learn, predict, or assimilate to novel actions (Cross et al., 2007).

**Figure 1:**
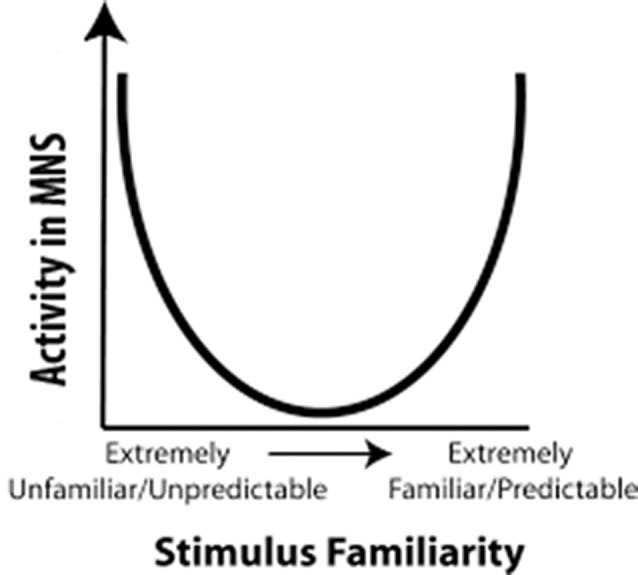
Familiarity Model: a representation of the predictability model proposed by Cross et al. (2012). The horizontal axis represents predictability of an action and the vertical axis represents activity in mirror neuron regions. In 2017, Cross and her colleagues updated this to a cubic model (Gardner et al., 2017).

Cross and her colleagues utilized this predictive model for the MNS to build a model that used predictability as a metric to explain fMRI results (Cross et al., 2012; Diersch et al., 2013). In their U-shaped model, observed actions that are extremely high or extremely low in predictability activated the MNS most strongly, while observed actions that are moderately predictable activated the MNS the least (Figure 1). However, when considering the neural efficiency theory, one would expect that increased familiarity with an action would result in a more efficient use of neural resources while perceiving that action. Cross and colleagues (Gardner et al., 2017) recently tested this theory and proposed adjusting the quadratic U-shaped predicative model to a cubic model to account for changes in neural efficiency upon increased familiarity with an action (Gardner et al., 2017). However, as we will delineate in “Relation of the Value Model to Human Data” section, the U-shaped model cannot explain why individuals with stroke show more activity for observation of actions made with the paretic rather than the non-paretic hand (the non-paretic hand is highly predictable while the paretic hand is moderately predictable, Garrison et al., 2013). Nor can it explain why observation of an action made by an amputee’s residual limb (highly unpredictable) and a typical hand action (highly predictable) should differ in their activation levels (Liew et al., 2013). While the cubic model has more potential to explain these results, the shape of the model remains largely unknown, and thus it is difficult to determine where many moderately familiar stimuli (e.g., paretic hand) would fit in the cubic model. For a stroke patient, a paretic hand is somewhere in the middle for familiarity, and in the cubic model, since the shape of it is vague, somewhere in the middle can either be high MNS activity or low MNS activity. Furthermore, while it is likely that familiarity and expertise modulate shared circuits, it seems that other factors, such as value, valance, etc., are needed to further explain these results. Indeed, there is a need to understand shared circuits as broader networks that are tied with emotion processing, reward systems and other circuits along with the sensorimotor networks within which they are commonly seen.

## Toward an Explanatory Model

**Figure 2:**
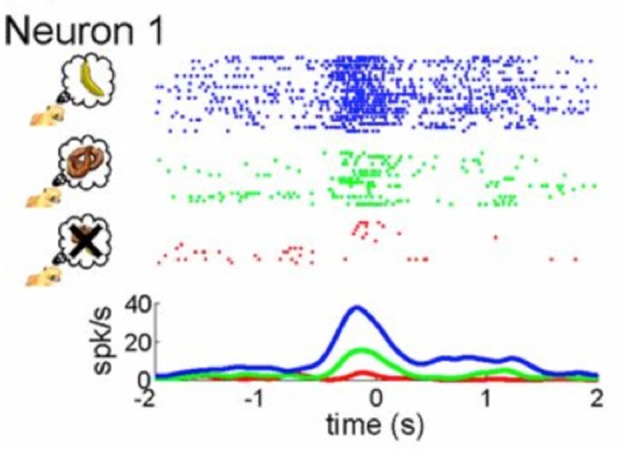
Mirror neuron activity is modulated by subjective value. As presented by Caggiano et al. (2012), the figure illustrates neural activity from single cell recordings of an exemplary mirror neuron in F5 of a monkey while it was presented with three different rewards: the most rewarding treat symbolized by a banana (blue coloring), a less relished reward indicated by the pretzel (green coloring) and an non-preferred food item represented by an “X” (red coloring). Taken from: Caggiano et al. (2012).

Recent neurophysiological findings may shed light on an underlying mechanism that modulates the MNS. Single cell recordings in monkeys indicate that subjective value drives activity in mirror neurons in F5 (Caggiano et al., 2012). That is, mirror neurons respond to the observation of actions that are valuable to the monkey, such as picking up a banana as compared to picking up a pretzel or another food item they are not fond of (see Figure 2). Even observation of actions that from the offset are arbitrary to a monkey, once paired with reward, elicit a stronger response in mirror neurons than actions that are not paired with reward (Caggiano et al., 2012). The researchers posit that through connections with reward circuits in the basal ganglia, mirror neurons are especially attuned to observation of actions that are subjectively valuable to the observer (Caggiano et al., 2012). These findings are reminiscent of findings from a previous study by Platt and Glimcher (1999) showing that activity in the lateral intraparietal area (LIP), a brain region involved in transferring visual information to motor actions in the monkey as well as a region later shown to have mirror neurons (Shepherd et al., 2009), is correlated with expected value. Here, we define value as the subjective importance, worth or usefulness of something. Subjective value can be modulated by reward, learning, valence, motivation and social context.

While the latter studies indicate that reward affects activity in the MNS, the direct neural pathways between monkey area F5 and prefrontal reward regions remain unknown. Previous studies indicate reward modulates neuronal activity during goal-directed actions in many parts of the brain, including mid-brain (ventral tegmental area [VTA], substantia nigra [SN]), basal ganglia and prefrontal cortex (Schultz, 2000; Schultz et al., 2000). This modulation is facilitated by the neurotransmitter dopamine, produced in the VTA, which is known to be involved with processing both natural and conditioned rewards. Interestingly, ventromedial regions of the VTA and substantia nigra compacta (SNc) may be involved in coding value, while dorsolateral regions of the SNc may be involved in coding motivational salience (Matsumoto and Hikosaka, 2009). From the VTA, dopamine signals are most strongly projected to the ventral striatum/nucleus accumbens (NAc). The NAc in turn is thought to encode reward signals from the VTA. While striatal neurons are thought to be involved in reward learning, they do not seem to encode the specific reward. By contrast, orbital and medial prefrontal (OMPFC) neurons seem to process the specific nature of the reward (Schultz, 2000; Schultz et al., 2000). In the vmPFC, they may also be involved in the assessment and assignment of the personal value of the stimulus (Kim and Johnson, 2013).

**Figure 3:**
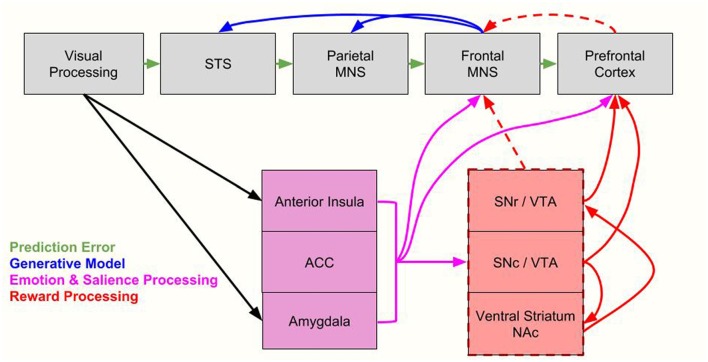
Integrating the proposed value-driven model with the Bayesian model of mirror neuron system (MNS) processing. Following processing in visual brain regions, information flows along established MNS pathways (Iacoboni, 2005) as well as along emotion processing/salience regions (anterior insula, anterior cingulate cortex [ACC], amygdala) and reward processing regions (substantia nigra reticulate [SNr], substantia nigra compacta [SNc], ventral tegmental area [VTA], ventral striatum/nucleus accumbens [NAc]). Here we integrate Bayesian models, which include prediction error signals (green arrows), and generative model processing (blue arrows, Kilner et al., 2007), with emotion and salience processing (pink arrows) and reward processing (red arrows). Indirect reward processing is depicted with dashed red lines. All of these processes modulate the MNS. The parietal MNS is thought to include the posterior parietal cortex (PPC) while the frontal MNS is thought to include the ventral premotor cortex (vPMC) and the inferior frontal gyrus (IFG). STS = superior temporal sulcus. For brevity, we don’t include every region of salient, emotion and reward systems, only primary nodes most likely to be directly related to the MNS. We also note that components of the reward system also process saliency, as discussed in “Toward an Explanatory Model” section.

While OMPFC neurons encode value, they are not modulated by the location in space a reward is given or the motor response associated with a reward. This is in contrast to other value processing brain regions, in which value modulates activity related to sensory or motor processes (Conen and Padoa-Schioppa, 2016). Thus indirect reward processing could modulate sensorimotor regions. For example, the anterior cingulate cortex (ACC) represents quantitative reward prediction errors (Amiez et al., 2006; Matsumoto and Hikosaka, 2007; Seo and Lee, 2007) especially of actions (Matsumoto and Hikosaka, 2007). Thus, its activity is closely tied to action selection and it may therefore be an important component for action observation as well. The anticipation of a large reward also triggers strong neuronal activation patterns in motor networks (Roesch and Olson, 2003, 2004, 2007; Wallis and Kennerley, 2010). The frontal eye fields, and the premotor cortex modulate attentional resources using the reward signal (Wallis and Kennerley, 2010) as do parietal regions (Platt and Glimcher, 1999). With regards to the premotor cortex, the study by Caggiano et al. (2012) on mirror neurons indicate this is true regardless of whether the reward is for the self or for another person. Again though, the direct pathways between mirror neuron regions and reward processing regions remains to be further explored, with current data showing indirect pathways to motor areas (Haber et al., 2000). As we delineate in Figure 3, such indirect pathways could allow not only reward signals to modulate the MNS, but also other factors such as salience, emotion and other cognitive processes. With regard to salience, while parts of the striatum code for this factor (Matsumoto and Hikosaka, 2009) and saliency is an important factor for processing one stimulus over another, it should be distinguished by motivational value, which is a separate factor. That is to say, all valuable stimuli may be salient (and thus chosen for further processing), but not all salient stimuli are valuable.

In our view, while the Kilner and Friston’s prediction model (also utilized by Cross et al., 2012) is an elegant one, and provides an excellent framework to understand the MNS, there is a need to understand the MNS circuit with broader networks that are tied with emotion processing, reward systems, saliency and other networks within which they are commonly seen (Caggiano et al., 2012). Here, we specifically propose a modification to the model such that preferred predictive models are those that are meaningful and valued. Indeed one study found increased activity in the caudate, as well as typical MNS regions, when participants had to predict another person’s future actions from more ambiguous prior observed actions, and the authors argued that ambiguous actions which need to be learned may be tied with more reward processing (Diersch et al., 2013). Pairing a value model with the prediction models may outcome as a tetrahedron model that combines the cubic model of Gardner et al. (2017) and adds to the dimension of value-driven modulation, though such a model is probably still underestimating the complexity added by other dimensions, such as valence and saliency. The need for Bayesian theories of brain processing to incorporate value, emotion and valence processing has previously been expressed by other researchers (Joffily and Coricelli, 2013).

## Relation of the Value Model to Human Data

We extend this value-driven model from the monkey data to better explain discrepant activation patterns in multiple shared circuits in the human data, such that a unified model may explain reported activation patterns from previous studies as a function of value. Below we discuss findings from shared circuit research and explain their results from a value driven perspective. We focus here particularly on the MNS and pain matrix, as there is more data on these shared circuits than the others. Here we organize shared circuit studies along three categories: (1) observation of significant others; (2) observation of action familiarity; and (3) observations of actions to be learned.

### Observation of Significant or Insignificant Others

Depending on the situation, observing either a loved one (Cheng et al., 2010) or an enemy (Fox et al., 2013) in pain can be equally meaningful and valuable for the observer and elicit more activity in the underlying shared circuits (Cheng et al., 2010; Fox et al., 2013). For example, if you are being pursued by an enemy, seeing them trip over and crouch with pain is highly relevant information to process, perhaps more so than observing a neutral or liked individual in pain (e.g., your child). In other circumstances, observing your child slam their finger in a door is the most relevant information to process. Thus, the significance or valence of a relationship cannot be the key-determining factor, but rather the meaning and value of the observed experience. Similarly, watching someone of the same race or a different race can be equally valuable information to an observer depending on the background, situation and circumstance of the people involved. Therefore, while some have previously argued that shared circuits are more active for those “like me” (Molnar-Szakacs and Uddin, 2013) we argue instead that activation patterns may be better explained by the value of the observed action and actor to the self, which depends on goals, relationships, personal history, context, environment and motivational state. Such a model may be able to explain the data better than the “like me” model which is built purely on motoric or physical similarity and familiarity. This could explain why in some cases individuals very different from the self can activate the MNS (Aziz-Zadeh et al., 2012; Fox et al., 2013), while in other cases more similar others activate the system (Calvo-Merino et al., 2005, 2006; Bangert et al., 2006). Furthermore, we note that the “like me” model has an implicit value consideration. Thus, the “like me” model may be simplified by thinking instead in terms of value.

In human studies, the value assigned to others is often determined quantitatively by comparing the activity elicited by viewing close family members in contrast to strangers. However, the complexity of human relationships makes interpreting quantitative data in regard to the value of a given individual difficult. In studies comparing self vs. non-self faces, researchers typically find differences in the bilateral and right IFG (Uddin et al., 2005; Heckendorf et al., 2016). However, when comparing personally familiar vs. unfamiliar faces (i.e., personally familiar faces vs. famous faces or one’s own child vs. a familiar but unrelated child), findings often indicate activity in the anterior paracingulate cortex, posterior superior temporal sulcus and precuneous suggesting personal knowledge retrieval (Gobbini and Haxby, 2007). The majority of studies do not report MNS brain region activation when comparing familiar and unfamiliar faces (Ida Gobbini et al., 2004; Leibenluft et al., 2004; for review, with a few exceptions, see Natu and O’Toole, 2011). This may be because they focus on face perception rather than action observation. However other studies do find IFG activation; Ishai et al. (2002) reported IFG activation during familiar (famous) vs. unfamiliar face processing and Taylor et al. (2009) reported similar results when individuals viewed their partner’s faces compared to a stranger’s.

Discrepant findings and tasks make the synthesis of the results difficult to interpret. In some instances, it appears that the IFG is sensitive not only to self-recognition (Kaplan et al., 2008) but also to the perceived closeness or value, such as observing a partner or an important political or social figure (Shah et al., 2001; Ishai et al., 2002; Taylor et al., 2009). However, these studies are not sufficient to detangle the “like me” from a “value” model since information about value was not collected from participants. Furthermore, “like me” characteristics can be correlated with value in some situations making these variables difficult to detangle. That is, similarity may be confounded by value (the more similar, the more value; (Mitchell et al., 2011; Tamir and Mitchell, 2013). Perhaps a way to disentangle the value model from the “like me” model is to consider a study where you compare situations in which you judge characteristics of similar vs. dissimilar others as well as features you value. For example, you may be similar with another person in that you are both smokers, but you consider smoking to be a negative value. By contrast, you may be similar with another person in that you both have athletic physiques, and you value this physique very much. Future studies such as this need to be explored.

Nevertheless, dissimilar to the “like me” model, the value driven model could explain reported findings of increased activity in MNS regions for both familiar (Hadjikhani et al., 2007; Avenanti et al., 2010; Liew et al., 2011) and unfamiliar individuals (Qin and Northoff, 2011; Aziz-Zadeh et al., 2012). Furthermore it could explain discrepant findings such as why some reports indicate increased activity in MNS regions for human agents than robotic or non-human agents (Tai et al., 2004; Costantini et al., 2005; Engel et al., 2008; Chaminade et al., 2010; Miura et al., 2010; Shimada, 2010) while others find more activity for robotic as compared to human agents (Cross et al., 2012, etc.). With regard to familiar individuals, in a study by Losin et al. (2012), individuals observed and imitated an unfamiliar hand action performed by actors of different races. The researchers found that neural activity during imitation was modulated by race of the actor that participants were imitating. Specifically, more activity was elicited in Caucasian and Asian participants when observing African American actors than any other race, including their own. The authors suggested that perceived social status (i.e., African Americans having the lowest perceived social status; Fiske et al., 1999) might be reflected in neural activity during imitation. In other words, activity was modulated by the perceived value of the actor and not by similarity to the participant (“like me” model). A follow up study by the same group tested this theory by recruiting African American subjects to participate in the same experiment. Indeed, African American and European Americans activated MNS regions more when observing African American actors than when observing European Americans ones (Losin et al., 2014; see also Avenanti et al., 2010). The authors posit that social status, rather than racial similarity, is responsible for this racial modulation during observation. This theory of perceived social status supports a “value” driven model of the MNS such that perceived value of an individual is driving activity in this system. Finally, such a model would predict that socially relevant stimuli, like individuals facing toward the observer rather than away from the observer, should show modulation in the MNS (Kilner et al., 2007). We note that in these examples, it is possible that value can interact with saliency and both factors are important for increased processing in the MNS (see Figure 3).

### Observation of Action Familiarity

Calvo-Merino et al. (2005) reported that ballet dancers show increased activation in the MNS when watching ballet as compared to martial arts than those with no ballet expertise. Indeed, a number of studies indicate increased MNS activity for observed actions that are more familiar to the viewer or that the viewer has expertise in performing (Buccino et al., 2004; Järveläinen et al., 2004; Calvo-Merino et al., 2005, 2006; Cross et al., 2006; Wright et al., 2010). For example, when participants think they are observing the actions of another human being as opposed to a robot or inanimate agent, there have been reports of increased MNS activity (Wheatley et al., 2007) and motor priming. As we previously mentioned, this has led some people to propose that action familiarity drives activation in the MNS (see Figure 1).

Increased shared circuit activation for familiar actions is also observed in other sensory domains such as the auditory domain (Ricciardi et al., 2009). In a study by Ricciardi et al. (2009), congenitally blind individuals activated a premotor–temporoparietal cortical network in response to the sounds of actions (e.g., hammering). These regions overlapped both with MNS areas found in sighted participants in response to seeing or hearing an action, and with the brain response elicited by motor pantomime of the same actions. Furthermore, the MNS showed significantly greater activity to motor familiarity (actions previously performed by an individual) than to unfamiliar action sounds in both sighted and blind individuals.

However, familiarity with the action does not always correspond to more MNS activity. Some studies indicate no significant differences in key MNS regions when participants viewed human vs. robotic hand actions (Gazzola et al., 2007), or hands vs. geometric objects making goal directed actions (Ramsey and Hamilton, 2010). In fact some studies find increased MNS activation when observing non-human (e.g., robots) compared to human actions (Cross et al., 2012; Saygin and Stadler, 2012). Furthermore, it has been found that observing actions made by a limb that you do not have (e.g., a residual limb in an amputee) activates the MNS more than a limb that you do have (e.g., a hand; Liew et al., 2011). Thus, action familiarity cannot explain these discrepant results. However, in a value model, this discrepancy could be explained by looking at the value to the viewer behind the observed actions. In the first study discussed here by Calvo-Merino et al. (2005), it is likely that a ballet dancer would likely find observing ballet more “valuable” or meaningful than observing martial arts. Furthermore, learning the kinematics of a novel effector (e.g., a residual limb), or a novel agent (a robot) performing a dance, may be more meaningful. Furthermore, deciphering the underlying mechanisms of action understanding becomes more difficult when familiarly with actions and actors interact. The interplay between familiar and unfamiliar actions performed by similar or non-similar raced actors can elicit multiple neural systems as complexity increases. Activity in MNS and mentalizing regions (i.e., temporal-parietal junction [TPJ]) has been observed to increases when European American and Chinese participants view similar race actors performing actions compared to non-similar actors as well as when viewing unfamiliar hand actions compared to familiar actions in the same individuals (Van Overwalle and Baetens, 2009; Liew et al., 2011). Depending on the complexity of the situation and level of individual engagement, it is thought that multiple networks contribute differently to the understanding of actions (Liew et al., 2011). A “like me”, “familiarity”, or even a saliency model cannot explain the dynamic activation patterns seen in these studies, where a value driven model may be better able. The MNS activates with increased familiarity and unfamiliarity depending on the task and individual engagement. The value of the observed action does not necessitate that the individual be familiar with the actor or action.

Indeed, familiarity may have at least two effects: reduction of uncertainty (e.g., in action execution), and facilitation of automatic responses (e.g., motor repertoire for ballet dancers). Reduction of uncertainty interacts with value in that individuals prefer familiar contexts to unfamiliar ones (ambiguity aversion, discounting unfamiliar contexts due to entropy, Calvo-Merino et al., 2006; Gazzola et al., 2007). Thus, in some cases, higher activation in the MNS for familiar observed actions can be explained because it is more valued and because it activates automatically. We explore how novel actions may show increased MNS activity in the “Observation of Actions That Need to be Learned or Relearned” section.

### Observation of Actions That Need to Be Learned or Relearned

We extend “value” to also incorporate the need for motor or social learning. Clearly, if there is a need to learn an observed action, then there is more value in observing that action and we would expect increased activity in the mirror system. Thus, as mentioned earlier, individuals who have different bodies than ourselves (e.g., an individual with an amputated limb) are people whose bodies we need to learn more about. Hence, some results show that there is more MNS activity in typically developing individuals when observing individuals performing actions with amputated limbs (Liew et al., 2013). Furthermore, some studies have shown that observing actions with novel kinematics also yields increased activity in the MNS as compared to more familiar actions (Cross et al., 2012). Observed actions that are unfamiliar to individuals compared to familiar actions can preferentially engage the MNS when performed by nonconspecifics. These findings can be understood in terms of the value model. In some situations, understanding the actions and intentions of action regardless of who is performing it is more valuable when the goal of the action is important, such as when an action is being learned. Indeed, goal-directed actions recruit shared circuit networks more than ambiguous or non-goal directed actions (Iacoboni et al., 2005). Similarly, a stroke patient finds more value in observing an actor’s counterpart to the paretic hand rather than the non-paretic hand perform actions (Garrison et al., 2013). Again, it may be more valuable to understand the actions of a paretic hand over the non-paretic depending on the environment and situation.

## Relevance to Clinical Populations

To conclude the review, we speculate that in some instances, clinical cases may arise from abnormal function between value processing and sensorimotor processing. Here we will specifically consider data on individuals with autism spectrum disorder (ASD; Dapretto et al., 2006; Williams, 2008; Kana et al., 2011). Research suggests that the MNS may be important for understanding ASD for several reasons. First, extensive research has identified impairments in imitation and motor ability in individuals with ASD (Rogers et al., 2003; Mostofsky et al., 2006; Williams et al., 2006; Williams, 2008; Vanvuchelen et al., 2011). A recent meta-analysis and a systematic review concluded that motor coordination deficits, including but not limited to imitation, are present in individuals with ASD (even in studies that controlled for age and IQ), and that this should be considered a cardinal feature of ASD (Williams et al., 2004; Fournier et al., 2010). Based on evidence that the MNS is strongly involved in imitation (Koski et al., 2002; Heiser et al., 2003; Iacoboni, 2005), it has been proposed that individuals with ASD who have imitation deficits (Williams et al., 2004) may also have a deficit in the MNS (Dapretto et al., 2006; Williams et al., 2006; Oberman and Ramachandran, 2007), potentially along with deficits in other networks. Several studies support this hypothesis, showing differential MNS functioning in individuals with ASD as compared to typically developing individuals (Dapretto et al., 2006; Williams, 2008; Kana et al., 2011). However, a few behavioral studies have indicated no imitation deficits, or “hyper imitation” deficits, in high functioning individuals with ASD, and no differential MNS functioning in individuals with ASD (Hamilton et al., 2007; Press et al., 2010; Spengler et al., 2010). These discrepant findings can arise from a number of factors. Some of the latter studies used adult populations (Press et al., 2010; Spengler et al., 2010), which is problematic because the one study that examined the effects of age on the AON found increased activity in this system as a function of age in individuals with ASD (Bastiaansen et al., 2011). Other studies that failed to find between-group differences had small sample sizes (Avikainen et al., 1999) or used many ASD subjects with autism impairment scores in the normal range (Raymaekers et al., 2009). Heterogeneity of symptomology in ASD may also be an important factor to consider.

However, if a MNS deficit in ASD is true for at least a subgroup of individuals with ASD, it may be that impairments arise not just from abnormalities in the MNS regions, but also between interactions between the MNS circuit and the reward circuits. Indeed, a few studies have shown that individuals with ASD have an impairment in processing social rewards as compared to monetary rewards (Lin et al., 2012), and social as compared to monetary reward learning is associated with decreased frontostriatal activations in ASD (Scott-Van Zeeland et al., 2010). Furthermore, mutations in genes within the mesolimbic dopamine pathway have been linked to ASD (Hettinger et al., 2008; Staal, 2015), as have mutations in the dopamine transporter (DAT; Bowton et al., 2014). Indeed, the social motivation theory of ASD (Dawson et al., 2005) suggests that children with ASD do not find social stimuli rewarding. In other words, children with ASD do not value or find the same social information rewarding to the same extent as typically developing children. Therefore, if individuals with ASD do not value the same stimuli, then according to our proposed model, they would elicit less activity in MNS regions. In fact, the value driven model fits with results suggesting that in children with ASD, MNS activity and sociality traits (as measured by subsets of established social assessments, e.g., Social Responsiveness Scale; Constantino et al., 2003) are positively related (Dapretto et al., 2006). Children who have higher scores in sociality find social reward stimuli more valuable, and therefore would have higher levels of MNS activation. Indeed, a recent study in a non-clinical group of participants indicated that individuals with more autistic traits assigned less value to viewing human bodies in natural motion compared to human bodies in robot-like motion or non-human control motion (Williams and Cross, 2018). Future studies comparing the subjective value of social and nonsocial stimuli would be useful to determine the relationship between ASD, the MNS, and value.

In general, more research on MNS development is needed. While some researchers suggest that the MNS is present at birth (Lepage and Théoret, 2007), there is only indirect evidence of this. In adults, several studies posit that during action observation and execution, MNS activity may be detected by desynchronization of the electroencephalogram (EEG) mu rhythm recorded over motor regions (Muthukumaraswamy et al., 2004; Ferrari et al., 2012). Similar EEG results have been found with human infants (Southgate et al., 2010; Marshall and Meltzoff, 2011; Nyström et al., 2011) and monkey infants (Ferrari et al., 2012) during action observation and execution. Furthermore, data indicate that mu desynchronization during action observation is predicted by the infant’s own motor abilities (van Elk et al., 2008). Thus MNS activity is modulated by development (Woodward and Gerson, 2014). A previous study indicated that the mu rhythm in infants is sensitive to action goals (Southgate et al., 2010) and the researchers argue that this allows infants to predict the outcomes of other people’s actions (Southgate et al., 2009, 2010). However, the degree to which this activation pattern is related to social cognitive and reward functioning remains unknown. Future longitudinal studies may focus on integrating social behavioral data with neural data on the MNS during development. Indeed abnormalities in the development of social abilities has been correlated with less activity in the MNS (Dapretto et al., 2006), and a longitudinal study may better decipher if there is a directionality to the data. This would then have implications for clinical cases as well.

Finally, more work on structural cortical changes to MNS regions through development are needed. Specifically, the prefrontal cortical thickness progresses in an inverted U-shaped pattern from infancy to one’s mid-20s (Sowell et al., 2002). It is likely that these structural changes in the IFG reflect differing activation patterns in the MNS and can be linked with behavioral development. In addition, neural connectivity to other brain systems, such as the reward network, salience system and emotion processing systems are likely to change throughout development. This may be reflected in differences found between mu desynchronization in infants compared to adults (Marshall and Meltzoff, 2011). Linking the structural brain, EEG and behavioral data together will be important for better understanding the MNS and its connections with other neural systems (e.g., reward systems, saliency network, etc.) through development and potentially allow for a better understanding of clinical disorders.

## Conclusion

In sum, previous theories have yet to satisfy current literature findings. Here we propose adapting the “value-driven” model of the MNS proposed for monkeys to humans. Specifically, the role of the dopamine reward circuit and emotion processing in modulating MNS activity needs to be an important part of any future model of the MNS, or other shared circuits. After reviewing previous findings through the lens of this new model’s framework, we find to better explain discrepant activation patterns in multiple shared circuits in the human data.

Further research should be conducted to test if indeed this model can explain reported activation patterns from previous studies as a function of value. In order to test this model, one would need to be able to determine “value” of a stimulus to an individual. So logically then, to account for previous discrepant results, one would need to show that the cohorts in the different studies “valued” things differently. With a value driven model, one would predict that this type of comparison would result in a positive correlation between activity in the MNS, reward system and the value an individual contributed to a given condition or stimuli. Future studies are needed to further explore this hypothesis.

## Author Contributions

LA-Z conceived the review article and wrote the first draft of the manuscript with EK. GC edited and contributed to sections of the manuscript.

## Conflict of Interest Statement

The authors declare that the research was conducted in the absence of any commercial or financial relationships that could be construed as a potential conflict of interest.
